# Pressure-induced phase transitions and superconductivity in magnesium carbides

**DOI:** 10.1038/s41598-019-56497-6

**Published:** 2019-12-27

**Authors:** Sooran Kim, Kyoo Kim, Jahyun Koo, Hoonkyung Lee, Byung Il Min, Duck Young Kim

**Affiliations:** 1 Department of Physics, Pohang University of Science and Technology, Pohang, 37673 South Korea; 2 Department of Physics Education, Kyungpook National University, Daegu, 41566 Korea; 3 Max Plank–POSTECH/Hsinchu Center for Complex Phase Materials, Pohang University of Science and Technology, Pohang, 37673 South Korea; 4 Department of Physics, Konkuk University, Seoul, 05029 South Korea; 5 Center for High Pressure Science and Technology Advanced Research, Shanghai, 201203 China; 6 Department of Chemistry, Pohang University of Science and Technology, Pohang, 37673 South Korea; 7 Present Address: Korea Atomic Energy Research Institute (KAERI), 111 Daedeok-daero 989 Beon-Gil, Yuseong-gu, Daejeon, 34057 South Korea

**Keywords:** Electronic properties and materials, Superconducting properties and materials

## Abstract

Crystal structure prediction and in silico physical property observations guide experimental synthesis in high-pressure research. Here, we used magnesium carbides as a representative example of computational high-pressure studies. We predicted various compositions of Mg–C compounds up to 150 GPa and successfully reproduced previous experimental results. Interestingly, our proposed MgC_2_ at high pressure >7 GPa consists of extended carbon bonds, one-dimensional graphene layers, and Mg atomic layers, which provides a good platform to study superconductivity of metal intercalated graphene nano-ribbons. We found that this new phase of MgC_2_ could be recovered to ambient pressure and exhibited a strong electron-phonon coupling (EPC) strength of 0.6 whose corresponding superconductivity transition temperature reached 15 K. The EPC originated from the cooperation of the out-of-plane and the in-plane phonon modes. The geometry confinement and the hybridization between the Mg *s* and C *p*_*z*_ orbitals significantly affect the coupling of phonon modes and electrons. These results show the importance of the high-pressure route to the synthesis of novel functional materials, which can promote the search for new phases of carbon-based superconductors.

## Introduction

Carbon has attracted much attention as a key element of contemporary science and technology. It is even considered as the most promising platform for emergent energy materials, which might be able to replace the current main player, silicon, in the near future. Indeed, during the last few decades, this perspective has been realized through the experimental synthesis of novel low-dimensional carbon systems such as carbon nanotubes, fullerenes, and graphenes. Unlike other carbon-group elements, such as Si and Ge that possess only *sp*^3^ bonding at ambient conditions, carbon allows different orders of bonding such as *sp*^1^ and *sp*^2^ as well as *sp*^3^, which makes the phase diagram of carbon very complicated and can eventually lead to various interesting structures.

Furthermore, carbon compounds showing superconductivity have been intensively and extensively studied. Hannay *et al*., first reported graphite intercalation compound superconductors (GICs) with the superconducting critical temperature (*T*_*c*_) < 1 K (C_8_*A*, *A* = K, Rb, Cs)^1^. The recent discovery of YbC_6_ (*T*_*c*_ = 6.5 K), CaC_6_ (*T*_*c*_ = 11.5 K) promoted an interest in GICs, and various GICs have been since reported^2–7^. CaC_6_ produces the highest *T*_*c*_ of 15.1 K in GICs under the pressure of 7.5 GPa^8^. A graphene-nanoribbon structure with Ca, CaC_2_, was predicted to exhibit superconductivity with a *T*_*c*_ of 9.8 K at 95 GPa^9^.

It is believed that the phonon mediates the superconductivity in the metal-intercalated carbon superconductors, and the electron-phonon coupling (EPC) is the underlying mechanism of their superconductivity^10–12^. Therefore, analyzing the phonon modes is an essential step to explore the mechanism of the superconductivity. Roughly, two important vibration modes exist: One is a high-frequency in-plane mode, and another is a low-frequency out-of-plane mode. The EPC constant *λ* = $N(0){D}^{2}/M{\omega }_{ph}^{2}$ can be enhanced by the large *D* and low energetic *ω*_*ph*_, where *N*(0) is the density of states (DOS) at Fermi level (*E*_*F*_), *D* is the deformation potential that is related to coupling between the electron and phonon mode, *M* is the effective atomic mass, and *ω*_*ph*_ is the phonon frequency. Despite the large *D* of the in-plane modes, *λ* is small because of the highly energetic *ω*_*ph*_ of the in-plane vibrations in the denominator^10^. The interlayer state by intercalant atom plays an important role in promoting the *D* of the out-of-plane mode and drives the superconductivity in GICs^10,11,13,14^. However, the coupling to out-of-plane vibrations in GICs is limited because too small a distance between the intercalant atom and graphite could be unfavorable for superconductivity^10^. Therefore, increasing the coupling between the in-plane phonon modes and charge carriers can be another route to obtain high *T*_*c*_ in graphene-analogue structures. MgB_2_ with a *T*_*c*_ of 40 K is a representative example with a huge coupling of the in-plane mode to *σ* state of boron orbitals^15,16^. Doped-picene, which is a hydrocarbon superconductor with *T*_*c*_ of 18 K^17^, is reported to show that intramolecular in-plane carbon vibrations can contribute to the enhanced electron-phonon coupling^18^.

Mg–C compounds could be promising superconducting materials because various carbon-bonding motifs can exist in magnesium carbides^19^. Recently, new phases of Mg_2_C and *β*-Mg_2_C_3_ were reported by experimental and theoretical cooperation^19,20^. MgC_2_ crystallizes in a tetragonal structure with the space group *P*4_2_/*mnm*. It contains a C2 dumbbell structure, similar to that of CaC_2_^21^. Srepusharawoot *et al*. proposed a new phase of MgC_2_ containing the pentagon structure using *ab initio* random searching^22^. The pentagon structure is energetically more stable than the dumbbell structure according to their calculations, which implies that the dumbbell structure can be a meta-stable structure of MgC_2_. The dumbbell and pentagon structures are both an insulating phase^22,23^. Furthermore, Wang *et al*., reported the structural evolution and the superconductivity of MgC_2_ under pressure using first principles calculations^24^. The calculated maximum *T*_*c*_ for orthorhombic *Cmcm* and monoclinic *C2/m* structures are 11.3 K at 4 GPa and 7.1 K at 9.6 GPa, respectively. Therefore, it is worth studying the possibility of superconductivity at ambient pressure and the origin of the high *T*_*c*_ further.

In this paper, we systematically study Mg–C compounds under high-pressure using first-principles calculations. First, we perform crystal structure searching to find unprecedented compounds, and to verify existing experimentally-known compounds. Then, we investigate phases of MgC_2_ under pressure up to 150 GPa. The metallic phase of MgC_2_ is obtained and stable even at ambient pressure, which exhibits superconductivity with a *T*_*c*_ of 15 K. The large *λ* originates from the cooperation of both couplings of the out-of-plane and in-plane mode to charge carriers. Geometry confinement plays an essential role in enhancing the in-plane vibration contribution to the EPC.

## Results

### Crystal structure searching under pressure

**Figure 1:**
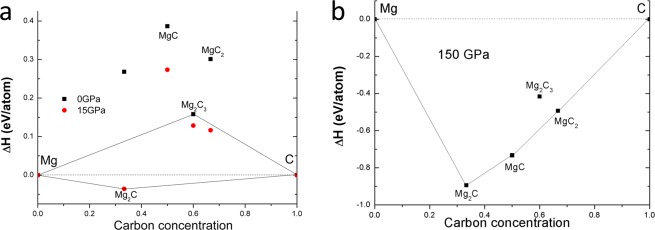
Crystal structure searching results. Convex hull (**a**) up to 15 GPa (**b**) at 150 GPa.

Figure 1 shows computational structure-searching results of Mg–C compounds at various pressures. At ambient pressure in Fig. 1a, the compounds are energetically unstable while two experimentally meta–stable compounds of MgC_2_ and Mg_2_C_3_ are close to the stability line (horizontal dashed lines at zero). At 15 GPa, Mg_2_C is stable with an anti-fluorite type structure, which has been reported experimentally^20^. Kurakevych *et al*. stated that their synthesis condition was 15 GPa^20^. This is in excellent agreement with our computational prediction. As the pressure increases, the formation enthalpy of MgC_2_ decreases from ~0.3 eV/atom at ambient pressure to ~0.11 eV/atom at 15 GPa. At a high pressure of 150 GPa, MgC_2_ can be one of the stable stoichiometries, as shown in Fig. 1b. This stable structure at high pressure will be discussed later. The enhanced stability of Mg–C compounds under high pressure is consistent with a previous report^25^.

**Figure 2:**
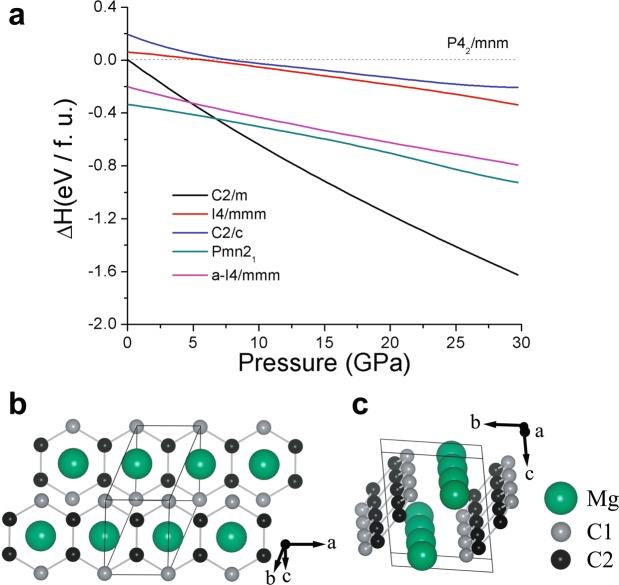
Enthalpy and the crystal structure of MgC_2_. (**a**) Enthalpies of the most stable MgC_2_ polymorphs as a function of pressure. The primitive cell of MgGNR structure at ambient pressure; (**b**) top view (**c**) side view with respect to zGNR.

**Figure 3:**
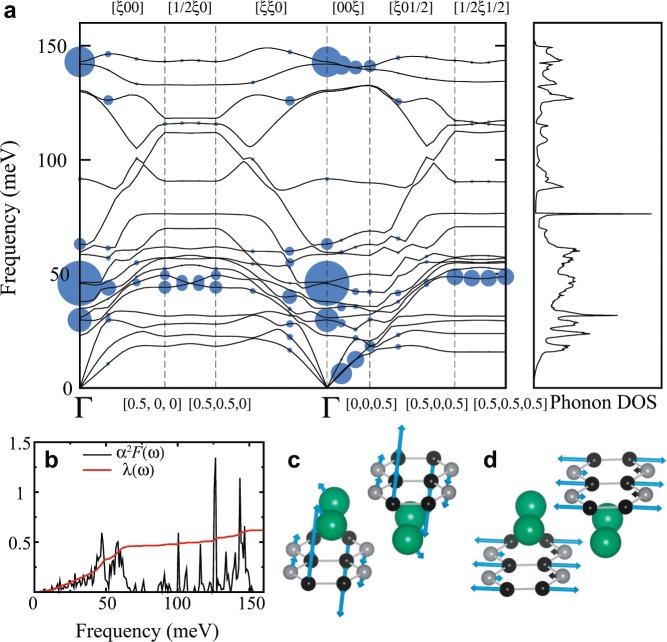
Phonon and EPC study on MgC_2_ at ambient pressure. (**a**) Phonon dispersion curve and total phonon DOS of MgGNR at ambient pressure. The size of each blue circle is proportional to the magnitude of *λ*_*qν*_. (**b**) Eliashberg function and *λ*(*ω*). Lattice displacements by two phonon modes, (**c**) **q**_1_ and (**d**) **q**_2_, provide the large contribution to *λ*. Blue arrows represent the displacements. The frequencies of **q**_1_ and **q**_2_ are 45.8 meV and 142.9 meV, respectively.

Figure 2a shows the formation enthalpy plot of MgC_2_ with a reference line exhibiting the previously observed dumbbell structure in the space group *P*4_2_/*mnm*. It is worth noting that some of our predicted phases show the lower enthalpy than that of the experimental structure. At ambient pressure, the Mg-intercalated graphite compound (*Pmn*2_1_) is predicted to be the ground state. However, when we tested its dynamical stability using *ab initio* lattice dynamics, it exhibits an imaginary acoustic phonon. Thus, it is unstable against decomposition. As it is a combination of graphene and Mg layers, it would be dissociated into individual graphene and Mg, which is manifested by the negligible charge transfer between C and Mg. Under pressure, we predict two structures; one is a-*I*4/*mmm* at low pressure <7 GPa, and another is *C*2/*m* structure at high pressure >7 GPa. The a-*I*4/*mmm* phase contains a C4 chain, which is more stable than the experimentally known phase at low pressure. Interestingly, *C*2/*m* consists of a single zigzag graphene nano-ribbon (zGNR) and Mg array. Hereafter, we refer to this *C*2/*m* phase as MgGNR. Figure 2b,c illustrates the top view and side view of the primitive cell of the *C*2/*m* structure. This structure has dynamic stability not only at high pressure but also at ambient pressure (See Fig. 3a), implying that the MgGNR phase can exist as a meta-stable structure at ambient pressure. The distances of C1-C2 and C2-C2 at ambient pressure are 1.53 Å and 1.45 Å, respectively.

### Electron-phonon coupling and superconductivity

Figure 3 shows the phonon dispersion curve and EPC properties of MgGNR at ambient pressure. The phonon dispersion curve and total phonon DOS without phonon softening in Fig. 3a indicates the dynamic stability of the structure. This phase is theoretically predicted to exhibit superconductivity. The superconducting parameters have been calculated using the Eliashberg EPC theory and the Allen-Dynes formula^26,27^;

1 $${T}_{c}=\frac{{\omega }_{log}}{1.20}\exp [\frac{-1.04(1+\lambda )}{\lambda (1-0.62{\mu }^{\ast })-{\mu }^{\ast }}],$$

where *α*^2^*F*(*ω*) is the Eliashberg function, ${\omega }_{log}=\exp [\frac{2}{\lambda }\int \frac{d\omega }{\omega }{\alpha }^{2}F(\omega )\log \,\omega ]$, and μ^*^ is the effective Coulomb repulsion parameter. The predicted *T*_*c*_ of 15 K in Table 1 is close to the maximum *T*_*c*_ of 15.1 K of existing GICs.

**Table 1:** Superconducting parameters of MgGNR. *N*(*E*_*F*_), *ω*_*log*_, and Θ_*D*_ are the DOS at *E*_*F*_, the logarithmic average phonon frequency, and the Debye temperature, respectively. *T*_*c*_ is obtained for effective Coulomb repulsion parameter μ^*^ of 0.1.

*N*(*E*_*F*_) (states/eV/f.u.)	*ω*_*log*_(*K*)	Θ_*D*_ (K)	*λ*	*T*_*c*_ (K) μ^*^ = 0.1
0.40	594.18	717.96	0.62	14.9

To explain this high *T*_*c*_, we analyzed the *λ*_*qν*_ and corresponding phonon modes. Interestingly, two regions give a large contribution to the total *λ* and Eliashberg function, as shown in Fig. 3a,b. The first region is around 30–60 meV at relatively low frequency, and the second one is around 140 meV. Two representative phonon modes at the Γ point produce large contribution to *λ*. The frequencies of the first mode, **q**_1_ and the second mode, **q**_2_, are 45.8 meV with *λ*_*qν*_ = 0.53 and 142.9 meV with *λ*_*qν*_ = 0.36, respectively. Figure 3c represents the lattice displacements by **q**_1_, which is the out-of-plane vibration mode by Mg and carbon displacements. This is consistent with existing GICs whose out-of-plane mode is usually chosen to explain their superconductivity. The in-plane vibration mode, **q**_2_, corresponds to the in-plane carbon displacement mode, as shown in Fig. 3d. The in-plane phonon mode is very similar to the phonon mode that mediates the superconductivity in MgB_2_^16^. Interestingly, MgGNR exhibits superconductivity induced by not only the out-of-plane mode but also the in-plane mode. These multiple phonon contributions to *λ* can produce the high *T*_*c*_ in MgGNR.

### Electronic structures without/with phonon modulation

**Figure 4:**
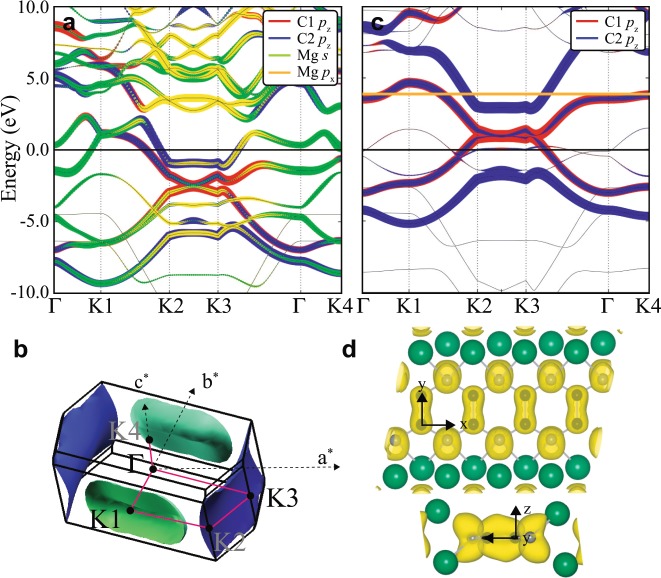
Electronic structure calculations at ambient pressure. Fat band structures for (**a**) MgGNR and (**b**) the hypothetical structure (HS). The orange line denotes the energy shift with respect to MgGNR. (**c**) The Fermi surface of MgGNR. The red line indicates the k-path of the band structure. (**d**) The charge density plot of MgGNR. The upper and lower ones are the top view and side view, respectively. *x*, *y*, and *z* represent the local axes. The charge density is integrated over −2 eV to *E*_*F*_ with an isosurface level of 0.047 e/Å^3^.

To investigate the properties of the predicted MgC_2_ and how **q**_1_ and **q**_2_ contribute to superconductivity, we performed electronic structure calculations without/with phonon modulations. The band structure of MgGNR in Fig. 4a shows the metallic property of the material. The *p*_*z*_ orbitals of C1 and C2, and the Mg *s* orbital are mainly observed near *E*_*F*_. The other *p*_*x*_ and *p*_*y*_ orbitals of C1 and C2 do not contribute near *E*_*F*_. Each Mg *p* orbital contributes less than the Mg *s* orbital. The crossing Mg *s* orbital corresponding to the interlayer state at *E*_*F*_ is consistent with that of superconducting GICs, which is proposed as a necessary condition for superconductivity^11,13,14^. Both the C *p*_*z*_ and Mg *s* bands are dispersive and show a strong hybridization feature except along the K2-K3 path. The *p*_*z*_ orbital of C2 is dominant and forms the flat band along the K2-K3 near *E*_*F*_. The flat band by C2 *p*_*z*_ shows the two-dimensional (2D) feature near *E*_*F*_. This electron-like *p*_*z*_ state along K2-K3 resembles the hole-like *σ* state along Γ-A in MgB_2_^28,29^ making a cylinder-shape Fermi surface from the 2D nature. Figure 4b represents the Fermi surface consisting of two sheets. The green sheet is derived from hybridization of the C *p*_*z*_ and Mg *s* orbitals, while the blue one is derived from the *p*_*z*_ orbitals of C2. Both Fermi surfaces are electron pockets. The blue Fermi surface by C2 *p*_*z*_ has a flattened cylinder-shape indicating its 2D character. The similarity to GICs and MgB_2_ suggests two different charge carriers; one is the electron in the Mg Fermi surface in green coupled with the out-of-plane phonon mode, and another is the electron in C Fermi surface in blue coupled with the in-plane phonon mode. This feature in the band structure calculations with deformed structures by each phonon mode will be discussed later. It is worth noting that the two-band superconductivity from the interband anisotropy is known to be important for MgB_2_. Compared to the isotropic calculation, considering the interband anisotropy increases the calculated electron-phonon coupling constant and predicted *T*_*c*_ from 0.77 and 22 K to 1.01 and 40 K, respectively^30,31^. Based on the similarity of MgC_2_ to MgB_2_, it would be interesting to investigate the anisotropic effect in MgC_2_ as future work.

To investigate the role of the Mg in the system, we set the hypothetical structure (HS) by removing the Mg atom from the MgGNR structure. The flat band along K2-K3 of HS in Fig. 4c is almost intact compared to that of MgGNR. The deformation of the bands by Mg orbitals mainly occurs in unoccupied *p*_*z*_ bands along Γ-K1-K2 and K3-Γ-K4. Another significant difference between MgGNR and HS is the position of *E*_*F*_. The band structure in HS is shifted upward by ~4  V with respect to that in MgGNR. MgGNR has the electron in the C2 *p*_*z*_ band whereas HS has the hole in the C2 *p*_*z*_ band, indicating that the role of Mg is electron doping to unoccupied *p*_*z*_ bands of GNR, forming the interlayer state at *E*_*F*_.

Figure 4d shows the charge density plot near *E*_*F*_. Note that *p*_*z*_ bands of C2 in zGNR are strongly deformed compared to those of graphite. The geometry confinement produces the bonding state between the *p*_*z*_ orbitals of two C2s. The top view of charge density resembles the *σ* bonding observed in MgB_2_. This deformation of the orbital shape can enhance the coupling of the in-plane phonon mode to the C2 *p*_*z*_ orbital. Therefore, the bonding state between the *p*_*z*_ orbitals of C2s increases the deformation potential *D* of the in-plane phonon mode, and provides the sizable *λ* despite its high frequency. This result also explains the difference and similarity between MgGNR and MgB_2_. Because MgGNR has two more electron per f.u. than MgB_2_, the C *p*_*z*_ orbital is mainly located at *E*_*F*_, whereas the B *p*_*x*,*y*_ orbitals are at *E*_*F*_ in MgB_2_^28,29^. The geometry confinement, however, produces the out-of-plane C *p*_*z*_ orbitals similar to the in-plane B *p*_*x*,*y*_.

**Figure 5:**
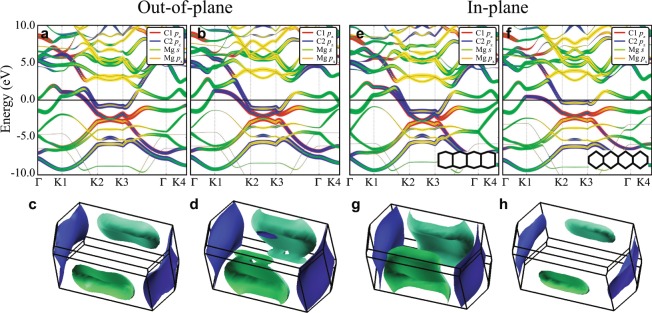
Band structures and Fermi surfaces of distorted structures with respect to the phonon modes. (**a–d**) the out-of-plane mode, **q**_1_ and (**e–h**) the in-plane mode, **q**_2_. Each mode has two band structures and two Fermi surfaces because of the two opposite directions of the corresponding displacements. The structures in (**a,c,e,g**) with the corresponding phonon mode in the positive direction while the structures in (**b,d,f,h**) with the corresponding phonon mode in the negative direction. The inset figures in (**e,f**) show the distorted zGNR by **q**_2_.

To check the effect of each phonon mode on the electronic structure, we calculated the electronic structures of the deformed structures by each phonon mode. For each mode, we considered two structures corresponding to two opposite displacements. Figure 5a–h show the band structures of the deformed structure with the out-of-plane mode, **q**_1_ and the in-plane mode, **q**_2_, respectively. The deformation by the out-of-plane phonon mode mainly occurs in the hybridized bands of Mg *s* and C *p*_*z*_ (the green Fermi surface), whereas the blue Fermi surface from *p*_*z*_ of C2 does not change much by the out-of-plane phonon mode. This suggests that the Mg electrons in the green Fermi surface are coupled with the out-of-plane mode and are the charge carrier for the superconductivity mediated by the out-of-plane phonon mode. It is consistent with GICs systems whose *λ* originates mainly from the out-of-plane mode, and the charge carriers are the intercalated-material electrons in the Fermi surface^11^.

On the other hand, the in-plane phonon mode produces a larger deformation in the whole Brillouin zone (BZ) as in Fig. 5e–h. Both green and blue Fermi surfaces are modified by the in-plane phonon mode. The blue Fermi surface by C2 *p*_*z*_ shows a huge modification in contrast to the case of the out-of-plane mode. It qualitatively shows that the deformation potential, *D*, of the in-plane mode is larger than that of the out-of-plane mode. The C2 *p*_*z*_ electron-like state also can be the charge carrier coupled with the in-plane phonon mode. Therefore, our results demonstrate that despite the high *ω*_*ph*_ of the in-plane vibration mode, it can contribute to the EPC with a large enough *D*.

In addition, we checked the HS structure (where Mg atoms are removed) with respect to the atomic displacements of each phonon mode. The electronic structures of the distorted structures are almost intact with respect to the out-of-plane phonon mode, while the electronic structures of the deformed structures by the in-plane mode are modified even without Mg states. This clearly suggests that the coupling between the electrons and the out-of-plane mode needs the interlayer state, whereas the coupling between the C *p*_*z*_ orbital and in-plane mode is not significantly affected by the interlayer state.

### Properties of MgC_2_ at 150 GPa

**Figure 6:**
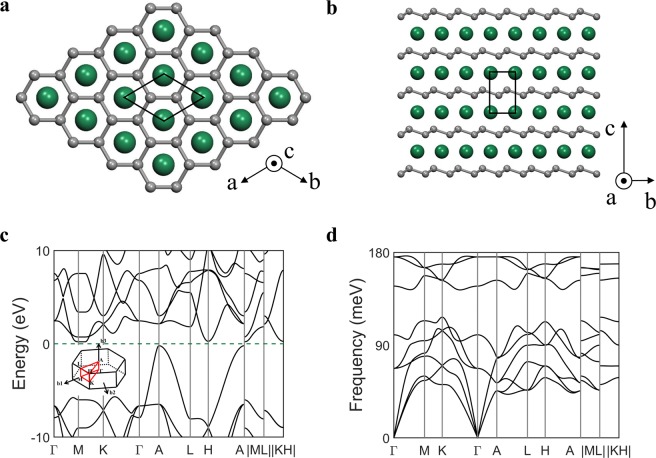
A high-pressure phase of MgC_2_. The crystal structure of MgC_2_ at 150 GPa with solid lines showing the unit-cell. (**a**) Top view and (**b**) side view. (**c**) Band structure of MgC_2_ and (**d**) stable phonon dispersion curve at 150 GPa. We chose the continuous Γ-M-K-Γ-A-L-H-A path followed by discontinuous M-L and K-H paths.

MgC_2_ undergoes a structural transformation to an ionic compound at around 150 GPa and is energetically stable, as predicted in Fig. 1b. The high-pressure phase of MgC_2_ possesses an intriguing geometry consisting of two-dimensional magnesium layer sandwiched by two buckled graphene layers, as shown in Fig. 6a,b, in good agreement with a previous theoretical prediction^24^. The carbon atoms consist of a hexagonal honeycomb lattice whose lattice constant of 2.42 Å (C–C distance, 1.48 Å) is comparable to the lattice constant 2.46 Å for graphene. The structure is similar to the BCS-type high-*T*_*c*_ superconductor, MgB_2_, except that the carbon atoms are not located on a plane due to a distortion along the *z* direction. It is a semiconductor with indirect–bandgap of 0.42 eV, as shown in Fig. 6c. The phonon dispersion curve without imaginary bands indicates its dynamical stability (See Fig. 6d).

**Figure 7:**
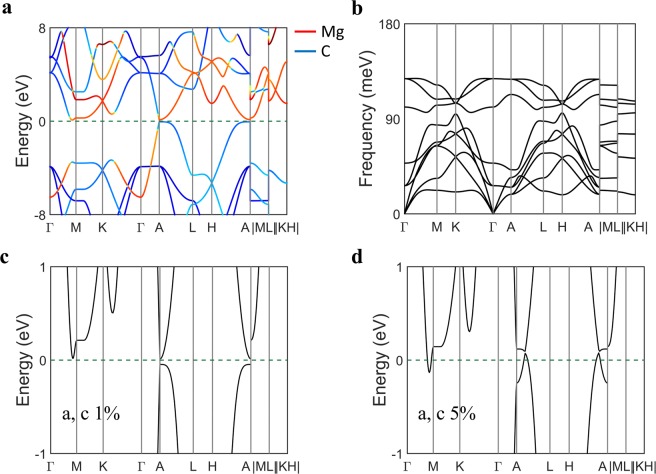
Electronic structure of the high-pressure phase of MgC_2_ at ambient pressure. (**a**) Fat-band plot of the band structure of MgC_2_ and (**b**) phonon dispersion curves. Zoomed-in band structure of MgC_2_ after (**c**) 1% and (**d**) 5% strain along the *a* and *c* axes. We chose the continuous Γ-M-K-Γ-A-L-H-A path followed by discontinuous M-L and K-H paths.

Figure 7a,b show the band structure and phonon bands of the high-pressure phase at ambient pressure, respectively. The phonon dispersion curve remains stable down to ambient pressure as in Fig. 7b, which implies its meta-stability at ambient conditions. Note that the band structure at ambient pressure in Fig. 7a shows that the conduction band minimum and valence band maximum become close to the *E*_*F*_ near *A* point. We further engineered the band gap by adjusting lattice parameters. A closer look at the bandgap in Fig. 7c exhibits a small gap opening at the *A* point with 1% expansion of *a* and *c* lattice parameters. The band gap is closed with 5% lattice expansion in Fig. 7d.

## Discussion

We computationally studied the high-pressure phases of MgC_2_. The phases show dynamic stability not only at high pressure but also at ambient pressure. From an application perspective, this feature is intriguing because the properties of this newly-designed materials can be exploited both at high and ambient pressures. The superconductivity of MgC_2_ is predicted to have a *T*_*c*_ of 15 K, which is high among similar carbon-based superconductors. The large *T*_*c*_ originates from the cooperation of the out-of-plane and in-plane phonon modes. The hybridization between Mg *s* and C *p*_*z*_ produces an interlayer state at *E*_*F*_, and the coupling with the out-of-plane mode. The geometry confinement deformed the C *p*_*z*_ orbital to the bonding state of C *p*_*z*_ orbitals, which is similar to the *σ*-bonding states in MgB_2_, and produces the large deformation potential of the in-plane mode. The graphene-nanoribbon superconductor, MgC_2_, shows the coupling mechanism between charge carriers and phonon modes observed in GICs and MgB_2_. Thus, we believe that metal-intercalated graphene-nanoribbon superconductors like MgC_2_ can be a model system to investigate the relationship between geometry confinement and superconductivity in low-dimensional systems.

## Methods

To investigate ground state structures of various Mg-C compounds containing up to four formula units under pressure, a structure searching algorithm USPEX^32,33^ combined with VASP was employed^34^. We conducted USPEX calculations up to 12 atoms in the simulation cell and set the maximum generation number to 50 and structure number to 60. The calculations were converged within the limit. The generalized gradient approximation of Perdew, Burke, and Ernzerhof (PBE) was used to describe the exchange correlation functional^35^. PAW pseudopotential^36^ was use in the USPEX-VASP calculations with energy cutoff 500 eV and 2 *π *× 0.01 Å^−1^ Brillouin zone grid of spacing to show a converged energy. After obtaining the structure at high pressure, the structure at ambient pressure was generated by the full relaxation of the atomic positions and the volume.

Harmonic phonon and Electron phonon coupling (EPC) calculations were conducted using Quantum Espresso software^37^ based on density functional perturbation theory^38^. We utilized ultrasoft pseudopotential and PBE functional for EPC calculations. EPC matrix elements were computed in the first BZ on a 6 × 6 × 6 q-mesh with a 24 × 24 × 24 k-mesh and 60 Ry energy cutoff. The convergence of EPC matrix elements was checked with a 12 × 12 × 12 q-mesh with a 24 × 24 × 24 k-mesh. To evaluate the integrated EPC constant (*λ*), we used the Gaussian broadening of 0.025 Ry.

The band structure calculations were performed with the full-potential linearized augmented plane wave band method implemented in Wien2k package^39^. Generalized gradient approximation (GGA-PBE) is used as the exchange-correlation.
